# Challenges facing providers of imported malaria-related healthcare services for Africans visiting friends and relatives (VFRs)

**DOI:** 10.1186/1475-2875-13-17

**Published:** 2014-01-09

**Authors:** Penny E Neave, Caroline OH Jones, Ron H Behrens

**Affiliations:** 1 Department of Community Health Development, AUT University, Auckland, New Zealand; 2 Department of Clinical Research, Faculty of Infectious and Tropical Diseases, London School of Hygiene and Tropical Medicine, London, UK; 3 Department of Disease Control, Faculty of Infectious and Tropical Diseases, London School of Hygiene and Tropical Medicine, London, UK; 4 KEMRI Wellcome Trust Research Programme, Kilifi, Kenya; 5 Nuffield Department of Clinical Medicine, Centre for Tropical Medicine, University of Oxford, Oxford, UK

**Keywords:** African VFRs, Imported malaria, Healthcare services, Migrant health

## Abstract

**Background:**

In many non-malarious countries, imported malaria disproportionately affects Africans visiting friends and relatives (VFRs). Most previous research has focused on understanding the knowledge, attitudes and practices of these travellers, but has not examined the quality of prevention, diagnosis and treatment services provided. The aim of this study was to understand the perspective of providers of malaria-related healthcare services to VFRs about factors impacting on the quality of these and to make recommendations about improvements.

**Methods:**

Thirty semi-structured interviews were conducted with practice nurses providing pre-travel health advice (n = 10), general practitioners (GPs) (n = 10), hospital consultants (n = 3), and community pharmacists (n = 7) working in areas of London with large African communities and a relatively high burden of imported malaria. A thematic analysis of the results was undertaken.

**Results:**

Time constraints in GPs’ surgeries and competing priorities, lack of confidence in issuing advice on mosquito avoidance, the cost of chemoprophylaxis and travel at short notice prevented the provision of adequate malaria prevention advice. Long GP waiting times, misdiagnoses, lack of disclosure by VFRs about recent travel, and the issue of where malaria treatment should be provided were raised as potential barriers to diagnosis and treatment.

**Conclusions:**

Some issues raised by respondents are relevant to all travellers, irrespective of their reason for travel. The challenge for healthcare providers to reduce the burden of imported malaria in VFRs is to provide services of sufficient quality to persuade them to adopt these in preference to those with which they may be familiar in their country of birth. Although no single intervention will significantly lower the burden of imported malaria, addressing the issues raised in this research could make a significant impact.

## Background

In many non-malarious countries, imported malaria disproportionately affects African migrants who visit friends and relatives (VFRs) in malarious countries [1,2]. Previous studies investigating why this is so have focused on understanding the knowledge, attitudes and practices of these travellers [3-5]. Little research has been undertaken to investigate the quality and effectiveness of malaria-related health services. One study which investigated the quality of pre-travel health services concluded that the provision of information in more deprived areas, in which larger Asian and African communities lived, was poorer than in more affluent areas [6], whilst another, carried out in East London where the majority of cases had visited African countries, reported misdiagnoses of malaria, especially by GPs [7].

The research presented here is part of a wider study, which explored the environmental, social, cultural, and structural factors that impact on the burden of imported falciparum malaria in Nigerian and Ghanaian VFRs living in London. The first part of the study found that despite the considerable heterogeneity in these communities, many were critical of malaria treatment services which are offered in the UK when compared to those provided in Nigeria or Ghana, and some were wary of using these services. This was also a finding from previous research carried out in the UK [3], and has been reported in a study conducted in the USA [8]. The aim of the research presented here was to understand the perspective of healthcare providers who provide malaria prevention advice, diagnosis and treatment in an area with a high incidence of imported malaria in Nigerian and Ghanaian VFRs about their practices, identify any barriers to their effective implementation and make recommendations about improvements.

## Methods

### Study setting and participant selection

The study took place in London between 2008 and 2011. Thirty participants took part in semi-structured interviews, each lasting between 30 and 45 minutes. The participants were: nurses who give pre-travel health advice (n = 10), general practitioners (GPs) who provide primary care for patients with suspected malaria (n = 10), Accident and Emergency (A&E) consultants who diagnose and treat patients (n = 3) and community pharmacists (n = 7).

A spreadsheet of laboratory-confirmed malaria cases was provided by the Malaria Reference Laboratory (MRL). This contained cases reported between 2001 and 2006 and it included patient’s postcodes. This information was used to identify the areas in London with the highest burden of this disease.

Initially, GP surgeries in postcode areas with a high incidence of imported malaria were contacted to recruit GPs. This proved unsuccessful and a second method was used. Five different professional acquaintances of the first author (PEN) gave contact details of seven potential GP participants who all worked at different surgeries. Each was contacted by email, and agreed to participate. Three additional GPs were recruited after their names were suggested by other respondents.

Seven practice nurses who give pre-travel health advice agreed to participate after their names were suggested by a GP who had taken part in the study. The name of the eighth practice nurse was suggested by a respondent who worked at a different surgery, whilst two responded to an email requesting participation in the study which was sent to all practice nurses working in one London Primary Care Trust (PCT) (Lambeth).

Two hospital consultants were professional acquaintances of PEN. They were contacted by email and agreed to participate. The third was suggested by one of these respondents. A fourth consultant initially agreed to participate, but subsequent attempts to contact him were unsuccessful.

A list of community pharmacists working in two London boroughs was obtained from the Internet. The same method of recruitment initially used to recruit GPs was employed. That is, areas within each of the two boroughs with the highest incidence of imported malaria were targeted. Pharmacies in these areas were selected at random, telephoned, and a request made to speak to the pharmacist. Where more than one pharmacist was available, the choice was also randomly made. Sixteen pharmacists were contacted and told about the study, and seven agreed to participate.

Ethical approval to carry out the study was obtained from the London School of Hygiene and Tropical Medicine’s ethics committee (reference number 5086).

### Data collection

A topic guide was used to direct the interviews. All respondents were asked initially about their level of experience. Questions asked to nurses and community pharmacists then focused on their experience of providing prevention advice and chemoprophylaxis to VFR travellers, and describing any barriers to delivering these services. Community pharmacists, as well as GPs and hospital consultants, were questioned about these issues with respect to malaria diagnosis and treatment.

Interviews were recorded using a digital recorder and transcribed verbatim. Transcripts of each interview were exported into the qualitative data analysis package NVivo version 7. A thematic analysis of the transcripts was undertaken.

## Results

Respondents worked in four of the 32 London PCTs. Employment and practice details of GPs and practice nurses are shown in Table 1.

**Table 1:** Sex, ethnicity, practice details and number of years employed in role for GPs and practice nurses

**ID**	**Sex**	**Ethnicity**	No of GPs in practice*	No of PNS in practice*	No of patients registered with practice*	% of patients of “Black African” ethnicity**	**PCT**	**Number of years working as a GP/practice nurse**
GP1	M	Caucasian	1-4	1-4	5,000-9,999	15-19	Lambeth	20-24
GP2	M	Caucasian	10-14	5-9	15,000-19,999	15-19	Greenwich	20-24
GP3	M	Caucasian	10-14	1-4	10,000-14,999	15-19	Lambeth	20-24
GP4	M	Caucasian	5-9	1-4	5,000-9,999	10-19	Greenwich	20-24
GP5	M	Caucasian	5-9	1-4	10,000-14,999	5-9	Lambeth	20-24
GP6	F	Afro-Caribbean	1-4	1-4	5,000-9,999	15-19	Southwark	10-14
GP7	F	Caucasian	5-9	1-4	5,000-9,999	5-9	Greenwich	10-14
GP8	M	Caucasian	1-4	1-4	5,000-9999	20-24	Lambeth	25-29
GP9	M	Caucasian	10-14	1-4	5,000-9,999	10-14	Lambeth	20-24
GP10	F	Indian sub-continent	5-9	1-4	15,000-19,999	15-19	Greenwich	1-4
PN1	F	Afro-Caribbean	1-4	1-4	5,000-9,999	15-19	Lambeth	5-9
PN2	F	Caucasian	10-14	5-9	15,000-19,999	15-19	Greenwich	5-9
PN3	F	Caucasian	5-9	1-4	5,000-9,999	10-19	Greenwich	15-19
PN4	F	Caucasian	5-9	1-4	5,000-9,999	5-9	Greenwich	25-29
PN5	F	Caucasian	1-4	1-4	5,000-9,999	20-24	Lambeth	5-9
PN6	F	Black Caribbean	10-14	1-4	5,000-9,999	10-14	Lambeth	1-4
PN7	F	Black Caribbean	10-14	1-4	10,000-14,999	5-9	Lambeth	10-14
PN8	F	Caucasian	5-9	1-4	15,000-19,999	15-19	Greenwich	10-14
PN9	F	Indian sub-continent	5-9	1-4	5,000-9,999	10-14	Lambeth	10-14
PN10	F	Black African	1-4	1-4	<5,000	5-9	Greenwich	5-9

*Data taken from the London Health observatory website [9].

**Data taken from UK census 2001 [10].

Two community pharmacists were male, five were female and they belonged to four different ethnic groups. All hospital consultants were male and of different ethnicities. All community pharmacists and hospital consultants had worked in this role for at least five years. No further details are provided in order to preserve the anonymity of these small groups of respondents.

### Operation of travel health clinics

Some practice nurses had more autonomy than others in the scheduling of the service. These individuals offered flexible clinic times, for example, one practice provided a service on one evening during the week until 20.00 hours. Some were disappointed when GPs had changed clinics’ opening times to those which they felt would be inconvenient to patients, and appeared to be operating within a system which they perceived as inadequate, but over which they had little control.

The working environment in GP surgeries was described as being extremely pressured. Patient waiting times to see a GP could be long. During the time of the study, the provision of some GP services attracted additional payments from the PCT, and travel health was not included in these. Thus, it was not always considered a priority. For instance, in one practice in a deprived part of London, the travel health service had been suspended for the previous six weeks, partly because of competing interests. Travellers were directed to non-National Health Service (NHS) funded travel clinics, for which they would be obliged to pay for a consultation.

Consultations typically lasted between 15 and 20 minutes although first time travellers might be allocated more time. This was often considered insufficient to persuade VFRs to purchase anti-malarials or to issue advice on how to avoid mosquitoes. Some nurses described their priority as “ticking all the boxes” to prove they had provided all the information required.

### Travel at short notice

A minority of VFRs sought pre-travel advice only a few days before travel, either from GP travel health services or at community pharmacies. The latter would direct them to GP surgeries as they could not provide chemoprophylaxis directly. Last-minute attendance by travellers for health advice was a major source of frustration for nurses, however, it was acknowledged this may be sometimes unavoidable for VFRs, for instance when they were attending family events, such as funerals.

### Mosquito control

Nurses and community pharmacists reported that VFRs rarely asked them for advice on how to prevent mosquito bites. Three nurses imparted this information routinely, as they were required to record on patient notes that they had done so. However, one felt there was no time available for a relaxed conversation. The others did not routinely give this advice. One explained this might be due to time pressures, whilst another expressed a lack of confidence in giving it. She, along with two others, gave the patients leaflets on mosquito bite prevention instead. Many nurses said VFRs seemed not to listen to their advice and their eyes appeared to “glaze over” when this aspect of malaria prevention was discussed.

### Cost of chemoprophylaxis

During the study period, subsidized malaria chemoprophylaxis was available for residents of three London boroughs. The majority of doctors and nurses were broadly supportive of this. Some considered it confusing however, and called for a London-wide policy.

Nurses not working in areas where chemoprophylaxis was subsidized believed the cost of drugs could be a deterrent to VFRs seeking pre-travel advice and starting chemoprophylaxis before travel. One estimated that half her African male VFR patients would not take chemoprophylaxis for this reason. This was not always linked to chemoprophylaxis being prohibitively expensive, but because many VFRs knew that other options were available:

*I think there’s still this thing about, oh well my family will have something, and it’s cheaper over there and I’ll get it from them, and when you explain, well, it might not be the right stuff, you know, it’s chloroquine based, it’s “Oh well it’s worked before, so why shouldn’t it work now”? And why do I want to give you, you know, or give the NHS fifty quid, or whatever?* (PN3)

For those who did want to purchase chemoprophylaxis before arrival in a malarious country, some VFRs travelling as a family were reported to purchase chemoprophylaxis for women and/or children only, with adult men telling nurses they would buy drugs upon arrival. Some asked for the names of suitable drugs they could purchase.

Three community pharmacists not working in areas where malaria was subsidized believed that whilst some VFRs might be unhappy with the cost, they would purchase it nevertheless. On a few occasions however, they reported that patients had not picked up prescriptions once they had become aware of their cost. All three hospital consultants considered the cost of chemoprophylaxis to be a deterrent for some patients, particularly for those travelling with children for a long period. One, working in an area where chemoprophylaxis was not subsidized was unaware that this policy existed.

### Diagnosis of malaria in primary care

GPs would consider malaria as a possible diagnosis when a patient presented with an unexplained fever or influenza-type symptoms and/or reported that they had recently travelled to a malarious country. Although some VFRs volunteered information about recent travel, this was not routine and the majority would ask about this as a matter of course.

They had a low threshold for arranging blood tests, explaining that it is better to “over test” than to “under test”, but acknowledged that patients may face a long wait in hospital phlebotomy services. The number of patients subsequently diagnosed with malaria varied by GP, with an annual range of zero to ten.

### Diagnosis of malaria in accident & emergency departments

Two hospital consultants estimated they saw about 200 patients with suspected malaria annually. The other had seen one or two suspected cases a week in the summer when he had previously worked in a general hospital. Approximately 80 to 90% of these patients were of African origin and 10 to 20% would have initially sought care from their GP. Some might have waited up to a week to do this. One consultant preferred patients with suspected malaria not to see their GP, but to immediately seek hospital care so treatment could be started promptly if necessary. After initial assessment and a blood test, patients might wait for several hours in A&E for a diagnosis.

One consultant cited occasions when GPs had not considered malaria as a potential diagnosis. However, a GP respondent explained the difficulties that they may face:

*A two, three year old boy who just had diarrhoea and vomiting. So many people have diarrhoea and vomiting aged two to three, and well, it’s just joining nursery isn’t it? What is it? And luckily I asked about fever, once he’d got a fever, “Oh, dear, it’s bad diarrhoea and vomiting then”. …but suddenly I pricked up my ears at that point, and mum wasn’t bothered at all about the fever, she was just worried about, she just felt for her little boy, who was vomiting all over the place, and I homed in on the fever, and said, “So he’s got a fever, have you been abroad recently, still thinking not necessarily malaria, it could be another tropical illness, and she said about the trip, and, actually, the boy hadn’t had malaria syrup and I sent that boy straight in and it was falciparum malaria, and I remember feeling lucky, feeling they were lucky, that I was lucky, that that was a close shave.* (GP3)

### Treatment of malaria

About 20 to 40% of patients tested were confirmed to have malaria, with most having been infected with *Plasmodium falciparum*, the most serious type of malaria. One consultant estimated about a third discharged themselves once they received their diagnosis, and typhoid (which he felt was considered by many VFRs to be a more serious disease) had been excluded. These patients would be given quinine and contacted a few weeks later to confirm that they had recovered. He wondered if treating malaria patients with uncomplicated falciparum malaria as outpatients, rather than routinely admitting them could become standard policy in his hospital, as it was in the hospital in which another respondent worked. The third consultant worked mainly in a paediatric setting, where all patients were admitted.

Community pharmacists reported that attempts to purchase treatments directly from them were uncommon, but that some VFRs became irate on learning that this was not possible.

## Discussion

Respondents in this study identified several barriers to providing efficient malaria-related pre-travel health services.

Time constraints, alongside a lack of confidence by some nurses, may mean that advice about mosquito bite avoidance is not covered in detail in travel health consultations. The apparent unwillingness of many VFRs to listen to advice may be a further deterrent. Nevertheless, mosquito avoidance is important. The efficacy of malaria chemoprophylaxis is approximately 90% but cannot be fully relied upon to prevent malaria. In addition, many VFRs who contract malaria travel without it [11]. This means that for some there is almost a total reliance on protection against mosquito bites to prevent malaria. Many members of African community groups are likely to have first-hand experience of the difficulties of avoiding mosquito bites, in contrast to some practice nurses, and engagement with these groups about how best to offer this advice would be worthwhile.

The cost of chemoprophylaxis was believed to be a deterrent to its use by many respondents, and other studies have reported similar results [12,13]. A recent study suggested that although there may be some reduction in malaria incidence by subsidizing malaria chemoprophylaxis, it may not substantially decrease this [14]. Cost may be a deterrent for some, but not for all, and other factors may be given equal or more consideration [15].

The solution adopted by one GP practice to overcome competing priorities was to direct travellers to privately funded travel health clinics during times they were not able to offer these services. An evaluation of the availability of NHS-funded travel health services, particularly in areas with large migrant populations, some of whom travel frequently is needed to ensure access to free travel health advice at a surgery close to their area of residence. Details of NHS-funded services should be given to all travellers by GP practices that opt out of providing them.

Another issue identified in the study was in providing travel health services for those seeking this shortly before travel. Although attendance at a travel clinic should be encouraged several weeks in advance of travel wherever possible so that vaccinations for other diseases can be given if needed, travel at short notice may be inevitable for some VFRs at certain times. Given the small number of respondents in this study, and as only one other study has identified travel at short notice to be an issue affecting the accessibility of travel health services for VFRs [3], it would be worthwhile to investigate further the proportion of imported malaria cases which can be attributed to difficulties in accessing pre-travel health advice at short notice.

Waiting times to see a GP remain an important problem for healthcare systems such as the NHS. An issue raised by one respondent was whether patients with suspected malaria should attend A&E directly instead and this question should be addressed by policy makers and clinicians. Misdiagnoses, even in an area where many healthcare professionals are familiar with malaria, highlight the need for continuing medical education, as the consequences of these can be serious. Determining the most appropriate location for malaria treatment for VFRs of African origin (within hospitals or as outpatients) is an increasingly important and topical issue, with recent European guidelines suggesting that patients such as VFRs with uncomplicated falciparum malaria may be managed as outpatients if carefully monitored [16]. Currently, different policies of malaria treatment are adopted in neighbouring London hospitals.

This study was small scale, and is one of a few to have addressed these issues. More research using different methodologies is needed to confirm the findings. The study was undertaken in an area with a large African migrant population, and the results may not be extrapolated to other areas. Furthermore, selection of respondents was not always random and there have been selection bias, as only those with an interest, and perhaps expertise in the topic chose to participate. However, given the difficulties in initial recruitment, a pragmatic approach was undertaken. The inclusion of a variety of groups of respondents, and limiting to two the number of participants suggested by one person ensured that a range of views was considered. Including GPs and nurses from the same surgeries meant their perspectives could be compared. Nearly all respondents had worked in this field for several years, and had a great deal of experience of the issues.

Some issues identified in the study may affect all travellers, irrespective of ethnicity and reason for travel. However, first-generation VFRs at least are familiar with a different way of managing malaria, where unlike in many non-malarious countries, access to malaria prevention, diagnosis and treatment is not controlled by healthcare providers. By using these methods however, they risk misdiagnoses and inadequate treatment.

Whilst no single intervention is likely to lower the burden of imported malaria in VFRs, because of the range of factors impacting on decision making and the heterogeneity within this population, focusing research only on the knowledge, attitudes and practices of VFRs risks ignoring the responsibility health service providers have in ensuring services provided are adequate and acceptable to the group most at risk of this disease. Addressing the issues identified in this study could have an important role to play in reducing the burden of imported malaria.

## Abbreviations

A&E: Accident and emergency; GP: General Practitioner; MRL: Malaria reference laboratory; PCT: Primary care trust; VFR: Visiting friends and relatives; GP: General practitioner.

## Competing interests

RHB is a member of the advisory board of Sigma Tau and has received research funding from Sigma Tau. The authors have declared that there are no other competing interests.

## Authors’ contributions

PEN jointly conceived the study, carried out the fieldwork, analysed the data and co-wrote the paper. COHJ jointly conceived the study, provided advice on the study design and analysis and contributed to writing the paper. RHB jointly conceived the study, provided expert clinical advice and contributed to writing the paper. All authors read and approved the final manuscript.

## References

[B1] Leder K Black J O’ Brien D Greenwood Z Kain K Schwartz E Brown G Torresi J Illness in travelers visiting friends and relatives: a review of the GeoSentinel Surveillance Network Clin Infect Dis 2006 43 1185 1193 10.1086/507893 17029140

[B2] Pavli A Maltezou HC Malaria and travellers visiting friends and relatives Travel Med Infect Dis 2010 8 161 168 10.1016/j.tmaid.2010.01.003 20541136

[B3] Morgan M Figueroa-Muñoz JI Barriers to uptake and adherence with malaria prophylaxis by the African community in London, England: focus group study Ethn Health 2005 10 355 372 10.1080/13557850500242035 16191732

[B4] Pistone T Guibert F Gay D Malvy D Ezzedine K Receveur MC Siriwardana M Barouze B Bouchaud O Malaria risk perception, knowledge and prophylaxis practices among travellers of African ethnicity living in Paris and visiting their country of origin in sub-Saharan Africa Trans R Soc Trop Med Hyg 2007 101 990 995 10.1016/j.trstmh.2007.05.009 17643457

[B5] Schilthuis HJ Goossens I Ligthelm RJ De Vlas SJ Varkevisser C Richardus JH Factors determining use of pre-travel preventive health services by West African immigrants in the Netherlands Trop Med Int Health 2007 12 990 998 10.1111/j.1365-3156.2007.01856.x 17697094

[B6] Chiodini J The standard of malaria prevention advice in UK primary care Travel Med Infect Dis 2009 7 165 168 10.1016/j.tmaid.2009.02.003 19411043

[B7] Ladhani S El Bashir H Patel VS Shingadia D Childhood malaria in East London Pediatr Infect Dis J 2003 22 814 819 10.1097/01.inf.0000086401.13592.79 14506374

[B8] Leonard L VanLandingham M Adherence to travel health guidelines: the experience of Nigerian immigrants in Houston, Texas J Immigr Health 2001 3 31 45 10.1023/A:1026610602073 16228800

[B9] London Health Observatory Practice profiles 2011 http://www.lho.org.uk/commissioning/PracticeProfiles.aspx

[B10] Office for National Statistics Census: local characteristics on ethnicity, identity, language and religion for output areas in England and Wales 2012 2011 http://www.ons.gov.uk/ons/publications/re-reference-tables.html?edition=tcm%3A77-50029

[B11] Smith AD Bradley DJ Smith V Blaze M Behrens RH Chiodini PL Whitty JM Imported malaria and high risk groups: observational study using UK surveillance data 1987-2006 BMJ 2008 337 103 106 10.1136/bmj.a120 PMC2453297 18599471

[B12] Hossain J Private prescriptions and malaria chemoprophylaxis BMJ 2008 337 a135 10.1136/bmj.a135 18599472 PMC2453247

[B13] Hoveyda N McDonald P Behrens RH A description of travel medicine in general practice: a postal questionnaire survey J Travel Med 2004 11 295 299 15544713 10.2310/7060.2004.19105

[B14] Neave PE Taylor S Behrens RH Does public subsidy of the cost of malaria chemoprophylaxis reduce imported malaria? A comparative policy analysis Malar J 2013 12 238 10.1186/1475-2875-12-238 23848986 PMC3723845

[B15] Neave PE Jones CO Behrens RH A review of risk factors for imported malaria in the European African diaspora J Travel Med 2010 17 346 350 10.1111/j.1708-8305.2010.00440.x 20920057

[B16] Askling HH Bruneel F Burchard G Castelli F Chiodini PL Grobusch MP Lopez-Velez R Paul M Petersen E Popescu C Ramharter M Schlagenhauf P Management of imported malaria in Europe Malar J 2012 11 328 10.1186/1475-2875-11-328 22985344 PMC3489857

